# Helical tomotherapy of spinal chordomas: French Multicentric, retrospective study of a cohort of 30 cases

**DOI:** 10.1186/s13014-017-0768-1

**Published:** 2017-01-31

**Authors:** Maxime Bobin, Christina Zacharatou, Paul Sargos, Véronique Brouste, Albert Lisbona, Marc-André Mahé, Georges Noël, Amandine Halley, Loïc Feuvret, Louis Gras, Stéphanie Hoppe, Bénédicte Henriques de Figueiredo, Guy Kantor

**Affiliations:** 1 Department of Radiotherapy, Institut Bergonié, Comprehensive Cancer Center, 229 cours de l’Argonne, 33076 Bordeaux, France; 2 Clinical and Epidemiological Research Unit, Institut Bergonié, Comprehensive Cancer Center, 229 cours de l’Argonne, 33076 Bordeaux, France; 3 Department of Radiation Oncology, Institut de Cancérologie de l’Ouest René Gauducheau, Comprehensive Cancer Center, Saint-Herblain Cedex, France; 4 Department of Radiotherapy, Centre Paul Strauss, Comprehensive Cancer Center, 3 rue de la Porte-de-l’Hôpital, BP 42, 67065 Strasbourg, France; 5 Department of Radiotherapy, Hôpital de la Pitié-Salpêtrière-Charles-Foix, Assistance Publique Hôpitaux de Paris, 47-83, boulevard de l’Hôpital, 75013 Paris, France; 6 Academic Radiation Oncology Department, Centre Oscar Lambret, Comprehensive Cancer Center, 3 rue Frédéric Combemale, Lille, France; 7 University of Bordeaux, Bordeaux Cedex, France

**Keywords:** Helical tomotherapy, Spinal chordomas, Survival rates, Local control

## Abstract

**Purpose:**

To evaluate the efficacy and toxicity of helical tomotherapy (HT) in the management of spine chordomas when proton therapy is unavailable or non-feasible.

**Methods and materials:**

Between 2007 and 2013, 30 patients with biopsy-proven chordomas were treated by HT in five French institutions. Information regarding local control (LC), overall survival (OS), progression-free survival (PFS) and metastasis-free survival (MFS) was collected. Clinical efficacy, toxicity and treatment quality were evaluated.

**Results:**

Two-year actuarial LC, OS, PFS and MFS were 69.9%, 96.7%, 61.2% and 76.4%, respectively. HT treatments were well tolerated and no Grade 4–5 toxicities were observed. HT permitted the delivery of a mean dose of 68 Gy while respecting organ at risk (OAR) dose constraints, in particular in the spinal cord and cauda equina.

**Conclusions:**

This multicentric, retrospective study demonstrated the feasibility of HT in the treatment of spine chordomas, in the absence of hadron therapy.

## Introduction

Chordomas are rare cancers, representing 1–4% of primary bone cancers, with a global incidence of 8.4 in 10 million persons [1]. Chordomas are aggressive tumors with a strong tendency to recur locally after surgical resection and with a known resistance to radiotherapy and chemotherapy [2]. Radical en bloc resection with healthy tissue margins is currently the preferred treatment as it delivers best local control (LC) rates. However, this procedure is rarely possible because of the proximity of the tumor to neurological structures (spinal cord, cauda equina, nerve roots) or infiltration in soft tissue and vasculo-nervous axis or digestive and urinary system vicinity [3].

High-dose radiation therapy (RT), in particular proton and carbon ion therapy, leads to an increase in LC of the disease [4–6]. Unfortunately, these techniques remain very costly and are not readily available. Helical tomotherapy (HT) is a RT technique that combines intensity modulated RT (IMRT) and image guided RT (IGRT), thereby allowing dose escalation to the tumor while reducing doses to adjacent organs at risk (OARs). HT has shown, in our experience, dosimetric advantages to other forms of IMRT as it allows for steeper dose gradients at the border of the tumor than other radiation delivery techniques.

We report here the results of a French multicentric, retrospective study in which we address the technical feasibility, treatment quality and toxicity of HT in the treatment of 30 spinal cord and sacral chordomas. National recommendations for clinical indication target volume and OAR definitions were previously detailed and published before this study [7, 8].

Primary objective of the study was to assess the LC of the disease. Secondary objectives were overall survival (OS), progression-free survival (PFS), metastasis-free survival (MFS) and clinical efficacy and toxicity of tomotherapy treatments.

## Methods and materials

### Ethics approval

This study was performed with the permission of the Consultative Commission of the Treatment of Information in Research for Health (CCTIRS, Comité Consultatif sur le Traitement de l’Information en matière de Recherche dans le domaine de la Santé) and the National Commission of Information Technology and Freedom (CNIL, Commission Nationale de l’Informatique et des Libertés). Institutional Board approvals were also obtained. The study was performed in the context of a national program, previously defined and published [7, 8].

### Patient selection

Between 2007 and 2013, 30 patients diagnosed with biopsy-proven non-metastatic chordoma of mobile spine and sacrum were treated with HT in 5 different comprehensive cancer centers (Institut Bergonié Comprehensive Cancer Center, Bordeaux *n* = 14; René Gauducheau Comprehensive Cancer Center, Nantes *n* = 7; Paul Strauss Comprehensive Cancer Center, Strasbourg *n* = 6; Hôpital de la Pitié-Salpêtrière-Charles-Foix, Paris *n* = 2; Oscar Lambret, Comprehensive Cancer Center, Lille *n* = 1).

### Tomotherapy

When planning radiation therapy, Tomotherapy offered advantages over other techniques including IMRT, VMAT for clues of conformity, homogeneity and coverage. Furthermore, it facilitated a better dose gradient compared to other techniques, allowing maximum reduction of the dose to OARs. Hence, it was chosen as the preferred IMRT technique and all participating centers used the same Tomotherapy equipment. HT was performed by a Tomotherapy accelerator (Accuray® Inc.) using IMRT and IGRT techniques and 6 MV photons. Treatment planning used a dose calculation algorithm based on the convolution of pre-calculated photon kernels [9] and a dose optimization algorithm based on $\chi$ ^2^ minimization [10, 11]. Daily Megavoltage Computed Tomography (MVCT) was performed for patient positioning. Magnetic Resonance Imaging (MRI) examinations were used to define the target volume. Margins from Clinical Target Volume to Planning Target Volumes (PTVs) used were 3 or 5 mm. Nine tomotherapy plans with a simultaneous integrated boost technique were delivered.

### Statistical analysis

The analysis of survival rates was performed using the STATA v.11 software (Stata Corp., College Station, TX). The median follow-up of the series was calculated according to the inverse Kaplan-Meier method [12], with the deaths being censored [13]. OS, LC, PFS and MFS were calculated using the Kaplan-Meier method. OS was based on the date of first histological diagnosis to the date of analysis or time to death. LC, PFS and MFS were based on the date of the start of the tomotherapy.

### Simulation of proton therapy plans

Proton dosimetry plans were calculated with the treatment planning system Eclipse v.11 (Varian Medical Systems, Palo Alto, CA). The fluence of spot-scanning beams was optimized using the algorithm developed by Lomax [7] and the dose was calculated with a pencil beam convolution-superposition algorithm [6]. As treatment planning with protons does not require the use of opposed beams as in the case of photon beam therapy, we tried to reduce the number of beams as much as possible. In a total of 29 proton dosimetries (28 patients, one with a separate boost), 17 proton plans had 2 fields, 8 plans had 3 fields, 4 plans had 4 fields and one plan had 6 fields. All proton plans had at least two fields to reduce the dose to the skin, a third field was introduced to compensate for the more complicated anatomies (for better OAR sparing), and finally, 4 or more fields were necessary in the case of osteosynthesis material within the target volume to further homogenize the dose distribution.

### Dosimetric data collection

All dosimetric data were collected according to the International Commission on Radiation Units and Measurements, ICRU 83 criteria [14]. For the PTVs, the conformity indices (CoI) of the 95% (CoI_95%_) and 98% (CoI_98%_) isodoses (volume of 95% or 98% isodose divided by PTV volume, respectively), and the homogeneity indices (HI = (D_2%_–D_98%_)/D_50%_, where D_x%_ is the dose covering x% of the PTV volume) were recorded. For the OARs, we noted the maximal doses to the spinal cord, medullary canal, cauda equina, sacral nerve roots, rectum, bladder and bowels (including small intestine and colon). Healthy tissue was the volume defined by the external contour excluding the PTVs and we calculated and collected its average dose (in Gy) and integral dose (in J). Dosimetric data were collected in the same way for tomotherapy plans and for proton therapy plans.

## Results

### Patient characteristics

Table 1 summarizes the characteristics of the patient cohort. Mean patient age at the start of the treatment was 62.7 years (range, 36.7–83.1 years). Eighteen men and 12 women were treated. Three patients had cervical, 5 had lumbar and 22 had sacro-coccygeal chordomas. Mean interval between symptom expression and anatomical or pathological diagnosis was 19.2 months (range, 0–175 months). The disease manifested as isolated pain in 18 patients (60%), vesical-sphincterian dysfunctions in 4 patients, a combination of the two in 2 patients and one sacral chordoma was detected as a result of sub-acute cauda equina syndrome; upon diagnosis, 35% of the 23 evaluated patients were WHO 0 (8 patients), 52% were WHO 1 (12 patients), 9% were WHO 2 (2 patients) and 4% were WHO 3 (1 patient) [15].

**Table 1:** Patient characteristics

Characteristic	*n*
No. of patients	30
Male	18
Female	12
Age during RT	
< 40 years	2
< 60 years	11
Location	
Cervical spine	3
Lumbar spine	5
Sacrum and coccyx	22
Presentation	
Primary	24
Locally recurrent	6
Follow-up (25 patients alive)	
< 12 months	2
< 24 months	6
< 36 months	8
Surgery	22
R0	5
R1	11
R2	5
Implants	10

Twenty-two patients (73%) had an operation, of which 10 (45%) had osteosynthesis implants. The remaining eight patients (27%) had only biopsy. Surgical resection was evaluated as R0 (no microscopic residual disease) in 5 patients (23%), R1 (microscopic residual disease) in 11 patients (50%), R2 (macroscopic residual disease) in 5 patients (23%) and not classified in one patient (4%). Six patients (27%) had second surgery evaluated as R0 for one patient, R1 for 2 patients and R2 for 3 patients. Two patients received neo-adjuvant treatment, one with Imatinib and one with a combination of Imatinib and Cyclophosphamide.

Furthermore, when our cohort was treated, proton therapy for spinal and sacral chordomas was not available in France. Referrals for proton therapy abroad were refused because of accessibility or delay issues and technical reasons related to localization. The reasons for refusal were often multiple for a single patient. Our patient cohort was not optimal for proton therapy as several patients were in bad overall condition, had a very large PTV (mean 610 cc) and/or osteosynthesis implants. Proton therapy is readily available for patients in better general condition, and in particular for patients that are younger than our patient cohort (children and young adults having priority for proton therapy as a general rule).

### Tomotherapy treatment

Radiation therapy was proposed for 12 post-operative patients (40%) with residual disease (microscopic residual R1 for 7 patients and macroscopic residual R2 for 5 patients), for 8 patients (27%) with not-operated primary tumor, for 6 patients (20%) with relapse tumor and for 4 patients (13%) with operated primary tumor without residual disease R0. The median interval between pathology and beginning of HT was 4.6 months (range, 1.7–93.7).

Dose was prescribed to the median of the PTV, according to the ICRU 83 recommendations [14]. OAR dose constraints conformed to published recommendations [7, 8, 16–19]. For technical reasons, the dosimetric treatment plans of 2 patients were not recovered and therefore the dosimetric analysis was performed on the remaining 28 patients. Ten patients had two PTVs: PTV1 was the macroscopic target volume prescribed a boost dose in addition to the dose prescribed to the microscopic target volume PTV2. The boost volume was defined on post-operative MRI by the residual tumor volume. The use of simultaneous integrated boost plans depended on the individual center’s practices. Eighteen patients had a single PTV (PTV1). Median prescribed doses for PTV1 were 68 Gy (range, 61.2–74 Gy) in 35 fractions (range, 25–39) with a median fraction dose of 2 Gy (range, 1.8–2.5 Gy) and for PTV2 they were 59.6 Gy (range, 46–66.5 Gy) in 31 fractions (range, 23–35) with a median fraction dose of 1.9 Gy (range, 1.8–2). All patients were able to complete their treatment. The total and fraction doses were center-dependent.

No specific adjuvant treatments were performed following HT. At relapse, patients received different salvage including surgery, RT and general targeted therapy (Imatinib, Sorafenib, and Gefitinib) or chemotherapy (Doxorubicin).

### Analysis of survival

**Fig. 1:**
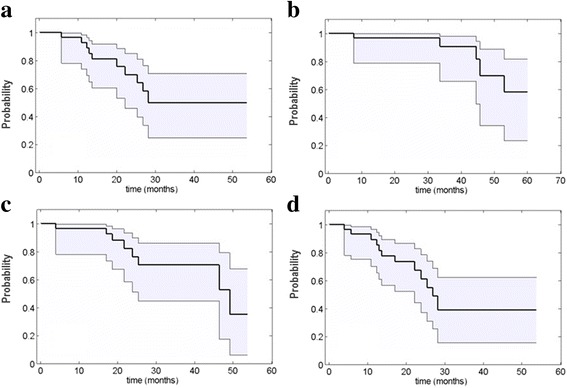
Survival probabilities with the 95% CI shown as the shaded area: (**a**) local control, (**b**) overall survival, (**c**) metastasis free survival and (**d**) progression free survival

The median follow-up time was 30 months (95% CI, 19.8–40). Ten patients (33%) developed a local failure. Actuarial 1- and 2-year LC rates were 92.7% (95% CI, 73.7–98.13) and 69.9% (95% CI, 46–84.8), respectively. At the time of the analysis, 5 patients died due to chordoma evolution. Two- and 3-year OS rates were 96.7% (95% CI, 78.6–99.5) and 90.6% (95% CI, 65.7–97.7), respectively. The 1- and 2-year MFS rates were 96.5% (95% CI, 77.9–99.5) and 76.4% (95% CI, 51.2–89.7), respectively (Fig. 1).

Eight patients (27%) developed a metastasis (in the order of highest to lowest frequency): bone, epiduritis, pulmonary/pleural, cutaneous or sub-cutaneous and muscular, inguinal and/or mediastinal adenopathies, liver, and one exceptional salivary glands metastasis confirmed by pathological examination.

### Clinical follow-up

All patients remained clinically stable during HT. At the time of diagnosis, 85% of the evaluated patients (23 out of 27) experienced pain, with 70% (7 out of 10) reporting pain ≤ 5/10 and three patients reporting pain between 6 and 7/10. At the completion of HT, 6 out of the 17 evaluated patients experienced no pain, 6 presented a pain from 1 to 5/10 and 5 reported a pain between 6 and 9/10. Retrospective data on analgesics (only available for a few patients) showed a minor increase to level 3 analgesics at the conclusion of treatment (from 2 out of 8 evaluated patients at diagnosis to 6 out of 14 evaluated patients at the end of HT).

**Fig. 2:**
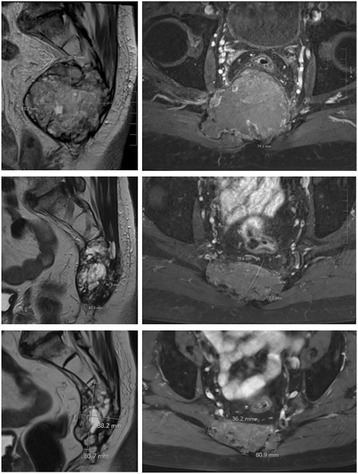
The case report of a 61 year-old male patient of the study, with an inoperable 8-cm sacrococcygeal chordoma treated by helical tomotherapy at a dose of 70 Gy. Treatment tolerance was excellent, with no acute or late toxicities and good efficacy on pain management. We note a regression of the tumor seen in MRI evaluations before helical tomotherapy (*top line*), at 1.5 years (*middle line*) and at 3 years (*bottom line*), in all directions mainly in the anterior-posterior plane from 83 mm to 38 mm (left: T2 sequences; right: axial T1 sequences after Gadolinium injection)

Post-operatively, of the 21 evaluated patients, 6 (29%) and 3 (14%) exhibited complications of Grade 2 and 3, respectively. These complications were not aggravated by HT. One year after HT treatment, 16 and 11% of the evaluated patients presented vesical sphincter disorders of Grade 2 and 3, respectively. An example of clinical and radiological case is shown in Fig. 2.

### Toxicity of tomotherapy

Radiation treatment toxicities are summarized in Table 2. There were thirty instances of acute Grade 1 and 2 cutaneous and digestive toxicities. Four early Grade 3 toxicities were reported. Three of them were cutaneous and one was digestive, involving mucoid diarrhea and dehydration requiring hospitalization. Two late Grade 3 toxicities were reported. One case of cutaneous necrosis and one case of radiation proctitis were observed. The latter was responsible for rectal bleeding which required hospitalization and endoscopic treatment by Plasma Argon. No radiation myelitis or radiation neuropathy cases were reported. No Grade 4 or 5 toxicities were observed. No definitive interruption of treatment due to toxicity was observed.

**Table 2:** Radiation treatment toxicities

	Acute toxicities			Late toxicities		
	Grade 1	Grade 2	Grade 3	Grade 1	Grade 2	Grade 3
Skin	9	9	3	5	1	1
GI^a^	9	3	1	0	0	1
Urinary	4	0	0	0	0	0
Neurological	1	1	0	0	2	0

^a^ *GI* gastro-intestinal

### Dosimetric data

**Fig. 3:**
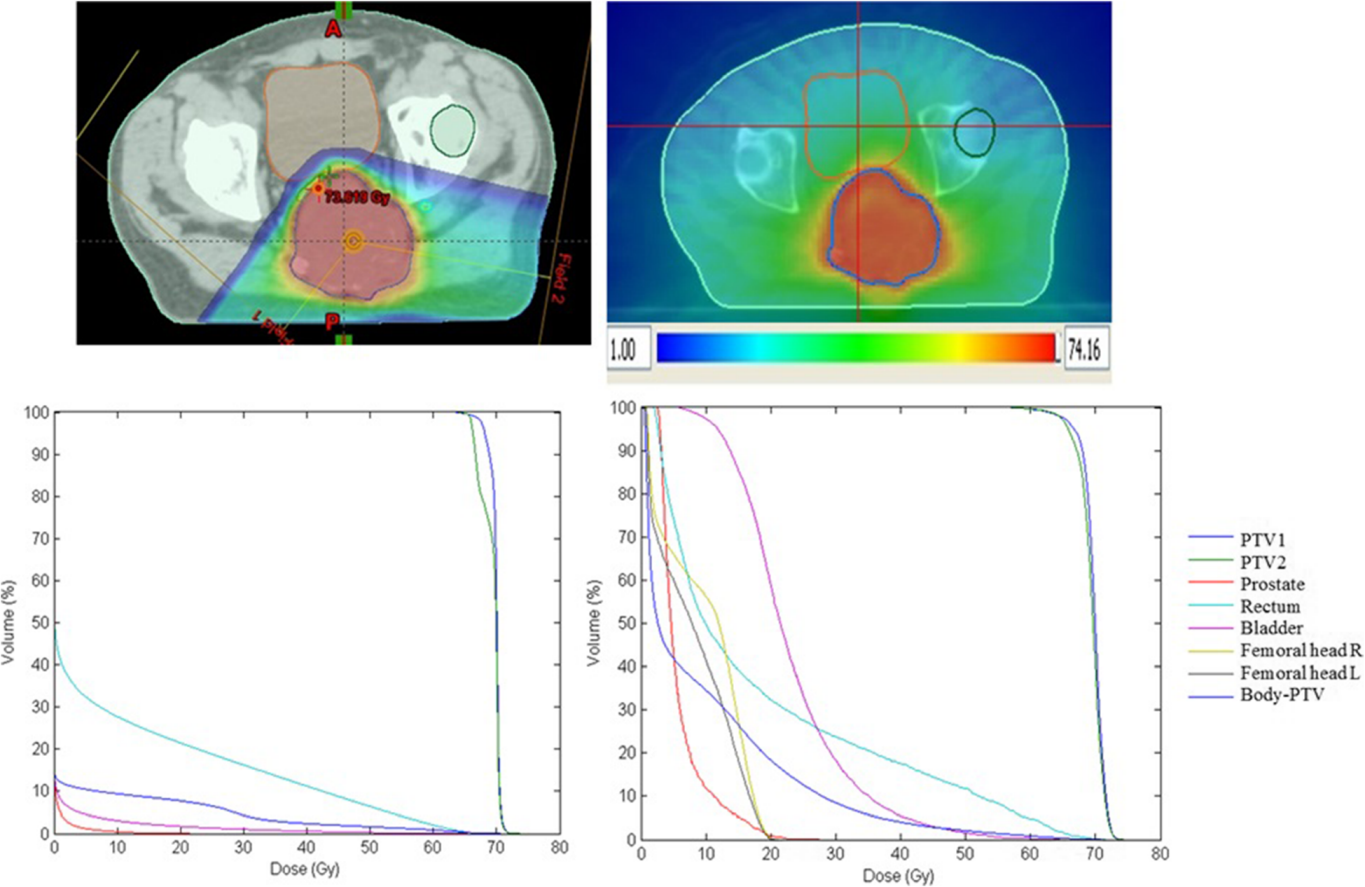
Comparison of a proton treatment plan (*left*) and a tomotherapy treatment plan (*right*) for the same patient. *Top panel*: the color wash indicates the volume covered by 1 Gy. *Bottom panel*: DVHs for the plans above

For PTV1, the mean volume was 610 cc (range, 7–2211), median CoI_95%_ was 1.25 (range, 0.83–4.13) for tomotherapy and 1.08 (range, 0.88–5.97) for proton therapy, median CoI_98%_ was 1.05 (range, 0.64–2.12) for tomotherapy and 0.93 (range, 0.71–1.62) for proton therapy, and median HI was 0.13 (range, 0.03–0.62) for tomotherapy and 0.07 (range, 0.03–0.91) for proton therapy. For PTV2, the mean volume was 659 cc (range, 27–1894), median CoI_95%_ was 1.49 (range, 0.89–4.12) for tomotherapy and 1.1 (range, 0.87–3.34) for proton therapy, median CoI_98%_ was 1.23 (range, 0.63–3.59) for tomotherapy and 0.98 (range, 0.42–3.15) for proton therapy, and median HI was 0.18 (range, 0.06–0.49) for tomotherapy and 0.09 (range, 0.03–0.27) for proton therapy. Median values of the minimal, maximal and mean doses to OARs are given in Table 3 for tomotherapy and proton therapy plans. In general, we observed that minimal and mean doses were lower in proton therapy. This is due to the lower number of beams that are needed for sufficient coverage of the target volume with the prescribed dose (Fig. 3).

**Table 3:** Median values of minimal, maximal and average OAR doses for tomotherapy (T) and proton therapy (P)

	D_min_ (Gy)	D_mean_ (Gy)	D_max_ (Gy)
Spinal cord, T	2.3	11.3	30.6
Spinal cord, P	0	15.1	42.8
Medullary canal, T	1.8	13.4	50.9
Medullary canal, P	0	8.1	57.7
Lumbosacral canal, T	1.8	25.0	49.4
Lumbosacral canal, P	0	13.3	51.9
Bladder, T	5.4	23.3	54.8
Bladder, P	0	0.1	15.4
Rectum, T	3.6	31.5	66.2
Rectum, P	0	13.2	69.1
Digestive system, T	1.1	17.4	63.4
Digestive system, P	0	1.5	64.2

**Fig. 4:**
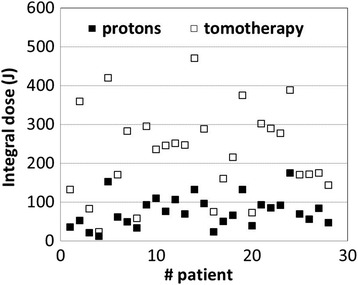
Comparison of integral doses for the 28 patients for tomotherapy vs proton therapy plans

Integral dose is significantly lower in proton treatment plans, as illustrated in Fig. 4. The median integral dose for the 28 patients considered in the dosimetric comparative study was 70 J for proton plans and 240 J for tomotherapy plans. Overall, proton dosimetries are superior to tomotherapy dosimetries, if one ignores the presence of metal implants, which tend to create hot and cold spots inside the PTV in proton plans. The only other exception we found to this conclusion were cases where the PTV completely encloses the spinal cord and in our experience, tomotherapy plans seem to better spare the spinal cord in those situations.

## Discussion

Different RT techniques have been evaluated for the treatment of spine chordomas and are listed in Table 4. In the case of photon beams, these included 3D conformal RT (3DCR) [20, 21], IMRT [22, 23] and stereotactic [24, 25] delivery. When compared to these techniques, hadron therapy with protons [4, 5, 26–30] or carbon ions [6, 31] showed better results in LC and survival.

**Table 4:** Results of the main series of radiotherapy of chordomas of the mobile spine and the sacrum

Beam	# Pts (location)	Dose Gy (RBE)	Dose/Fx Gy (RBE)	Median follow-up (years)	Local control at years	Global non-specific (specific) survival at years	Presence of metastasis	Reference
X	48: 23 (S) 20 (SB) 5 (MS)	50/40/24	2/1/8			54% at 5		Catton et al. [19]
						20% at 10		
X	26: 12 (S) 10 (SB) 4 (MS)	[30–66.6]	[1.8–2.5]			62% at 5		Cummings et al. [20]
						(83% at 5)		
						28% at 10		
X (IMRT)	34 (S)	66	[1.8–2]	4.5	27% at 5	70% at 5	9%	Zabel-du Bois et al. [21]
						(80% at 5)		
X (IMRT)	7 (MS)	66	[1.8–2]	1.45	65% at 2			Terezakis et al. [22]
X (SRS)	24: 10 (S) 7 (C) 4 (T) 3 (L)	18–24	[18–24]	2	95% at 4	67% at 4	12.5%	Yamada et al. [23]
X (SBRT)	18: 8 (MS) 7 (SB) 3 (S)	35	[6–8]	3.8	59.1% at 5	74.3% at 5		Henderson et al. [24]
^1^H +/−X	29 (MS)	76.6	1.8	7.3	84% at 5	87% at 5		Delaney et al. [4, 5]
	24: 19 (S) 2 (C) 1 (T) 2 (L)	77.4	[1.8–2.5]	4.7	90.4% at 3 79.8% at 5	78.1% at 5		Chen et al. [25]
	25 (MS, S)	70.4	1.8	2.65	73.3% at 5	64.3% at 5		Wagner et al. [26]
	26: 9 (C) 2 (T) 8 (L) 7 (S)	72	[1.8–2.5]	2.9	86% at 3	84% at 3		Rutz et al. [27]
	19: 12 (SB) 5 (C) 1 (L) 1 (S)	74	[1.8–2]	3.8	81% at 5	89% at 5		Rombi et al. [28]
	40: 16 (C) 4 (T) 10 (L) 11 (S)	72.5	[1.8–2]	3.6	62% at 5	80% at 5		Staab et al. [29]
12C	38 (S)	70.4	[4.4–4.6]	6.7	95% at 3 89% at 5	95% at 3		Imai et al. [6]
						86% at 5		
	17 (S)	70.4	[4.4–4.6]	4.1	100% at 5	53.3% at 5	14%	Nishida et al. [30]

*SB* skull base, *T* thoracic, *L* lumbar, *S* sacral, *MS* mobile spine, *C* cervical

The present study has a median follow-up of 30 months. A subsequent updating of data will allow a longer median follow-up and better knowledge of the effectiveness and longer-term toxicity of the technique. Our 2-year LC rate of 70% is inferior to the rates reported in the best series of proton or carbon ion therapy, which have 3-year and 5-year rates of 90 and 85%, respectively. Our 3-year OS rate of 90.6% is comparable to proton and carbon ion series (Table 4).

Twenty seven percent of the patients in our study presented a metastatic evolution of their disease. This result is comparable to other series in the literature with longer median follow-up times.

Conventional RT, up to 40–60 Gy doses, results in a 5-year LC rate of only 10–60% [3]. Terezakis et al. [23] reported that IMRT photon RT of 66 Gy resulted in a 2-year LC of 65% and a 2-year global survival of 79%, which is comparable to our results. Yamada et al. [24] used stereotactic RT in a single fraction of 24 Gy and obtained tumor regression or stability in 95% of patients at 2 years. Delaney et al. delivered up to 77.4 Gy relative biological effectiveness (RBE) to gross disease [4, 5] and found that proton therapy resulted in a 5-year and 8-year LC rates of 81 and 74%, respectively. Imai et al. [6] treated 38 inoperable sacral chordomas with carbon ions and observed a 5-year LC rate of 89%.

For various reasons, our LC rate is lower than reported in other series. Firstly, 73% of our cases were voluminous sacral localizations and this is associated with a less favorable prognosis. Secondly, 87% of our patients had residual disease when treated by HT. Staab et al. [30] reported that the 5-year LC rate decreased from 66 to 47% in the macroscopic residual cases. Similarly, Rutz et al. [28] reported that when the tumor residual exceeded 30 cc, the global- and progression-free survival results were statistically lower than in the case of smaller volumes. Thirdly, our patients had large target volumes (mean and median PTV values were 640 cc and 421 cc, respectively). Fourthly, osteosynthesis material was present in one-third of the patients. Out of the 10 local failure patients, one patient had a PTV larger than 610 cc and 2 patients presented osteosynthesis and 2 patients had both large PTV and osteosynthesis. Finally, we note that two-thirds of our patients were not eligible for proton therapy because of their altered clinical condition mainly due to the evolution of their disease. The retrospective nature of data collection did not allow us to obtain any specific clinical data which, therefore, limits the clinical description of patients.

Of note, osteosynthesis material could be an important limitation for proton therapy. For instance, Staab et al. [30] report on proton therapy showed the 5-year LC rate decrease from 100 to 30% when osteosynthesis material was present. Similarly, in Rutz et al. study [28], implants were also associated with a lower LC rate (*p* = 0.034). The authors proposed several factors that may explain the decrease in LC. Among major concerns, they noted that protons create important dose heterogeneity in the vicinity of the material with cold and hot spots, which requires a reduction of the fraction dose and/or total dose [28]. In addition, Verburg et al. [32] have demonstrated that osteosynthesis material can introduce uncertainties of up to 1 cm in proton range. Finally, osteosynthesis material complicates the delineation of volumes because of artifacts in the planning CT.

The clinical characteristics of our patients were comparable to those stated in the literature [1, 2]. Symptoms were dominated by pain, similar to other series [33]. We recorded partial or total pain alleviation in the available data. However, some data were missing as pain was not always reported. Vesical-sphincterian dysfunctions were not aggravated by HT.

In the majority of cases, HT was delivered without interruption due to toxicity. Only six cases of Grade 3 toxicity were recorded. In our cohort, we did not observe any Grade 4 or 5 toxicity, radiation myelitis or radiation neuropathy (including the roots of cauda equina). In general, more severe toxicities are reported in proton and carbon ion series. Delaney et al. [4, 5] observed three sacral neuropathies, following 76.6–77.4 Gy RBE and reported a complication risk of 13% for Grade 3–4 at 8 years. Chen et al. [26] found 8 sacral fractures, one secondary cancer, one foot drop, one erectile dysfunction and one loss of perineal sensation. Rutz et al. [28] reported four severe complications: one Grade 2 sensory neuropathy, one Grade 3 subcutaneous necrosis, one Grade 3 osteonecrosis and one Grade 5 secondary cancer. Imai et al. [6] described two late Grade 4 cutaneous reactions which required skin grafts, and six neurological complications, of which one was an incomplete transient aggravation of sciatic nerve paralysis. The small number of patients did not allow us to show significantly that the dose per fraction or the total dose were factors for local control, toxicity risk or relapse.

## Conclusion

This multicentric, retrospective study included an important series of patients, considering the rarity of the pathology and the cohorts discussed in other series. To our knowledge, this is the largest series of chordomas treated by HT. A longer follow-up is necessary to assess long-term survival and late toxicities. Hence, we conclude that HT permitted the delivery of high tumor doses with acceptable toxicities and can be used in the management of spine chordomas when hadron therapy is not possible.

## Abbreviations

3DCR: Three dimensional conformal radiation therapy
CI: Confidence interval
CoI: Conformity index
HT: Helical tomotherapy
IGRT: Image guided radiation therapy
IMRT: Intensity modulated radiation therapy
LC: Local control
MFS: Metastasis-free survival
MRI: Magnetic resonance imaging
MVCT: Megavoltage computed tomography
OAR: Organ at risk
OS: Overall survival
PFS: Progression-free survival
PVT: Planning target volumes
RBE: Relative biological effectiveness
RT: Radiation therapy
